# Surgery

**Published:** 1838-01

**Authors:** 


### SURGERY.

*On the Treatment of Erysipelas by raw Cotton.* By M. Reynaud.

Considerable experience appears to have established the utility of cotton in the treatment of burns. So convinced is the author of its value, that he employs it in all cases, in whatever degree they may exist: for he has found it to calm the pain instantaneously both in superficial burns of the skin and in those which occupy the whole thickness of the cutis, as well as to act beneficially in the subsequent process of the cure. Being struck with the efficacy of cotton in burns, and noticing the analogy which there exists between the inflammation of the skin produced by an incandescent body and that of erysipelas, M. Reynaud employed cotton in the latter disease, and the constantly beneficial results which followed its use led him to permit their publication.

The local treatment of erysipelas is, as M. Reynaud observes, very various, and based on no fixed principles. Some of the means employed give rise to considerable pain, others are, on various accounts, objectionable. In their place, M. Reynaud would substitute cotton, as a substance soft to the touch, of a moderate price, always at hand, easy to apply and unattended with pain in its application, and the action of which is so certain that the surgeon may at will extinguish or again excite the inflammation, by continuing or discontinuing the application. In erysipelas, as in burns, the cotton calms pain as it were by a charm; a mild and moist warmth takes the place of the itching, the formication, the sharp and biting heat which so much increase the pain; the swelling gradually diminishes, the redness disappears, the skin becomes flaccid and wrinkled, and without becoming covered with those furfuraceous scales, which characterize the termination of erysipelas, and which sometimes continue during a long period. All that separates are a few slight layers of epidermis, and this is speedily effected. The general excitement ceases with the local phenomena, the fever diminishes, and in simple cases the organic functions return to their normal state, without the necessity of any other treatment. A circumstance of great advantage in the use of cotton is, that it is equally fitted for, and produces analogous effects, in all forms of erysipelas, whether idiopathic or traumatic, whatever may be its situation, on the face, body, or limbs; whatever may be the depth of the tissues which are affected; for in the cases which are collected, are some of phlegmonous erysipelas greatly amended and others entirely arrested by the simple application of cotton. The result, M. Reynaud would thus explain,—that the cotton acts by exciting in the diseased part a moderate warmth, a sort of vapour bath which keeps up a constantly equable temperature, a proper degree of humidity, by keeping the diseased part from contact with air and light, two powerful excitants of the cutaneous system. Cotton does not suffice in all cases; no more than other remedies does it enable the surgeon to dispense with general means, but it lends to these a great assistance: it hastens the resolution, and when this termination cannot take place, it still serves to limit the inflammation, and to arrest its progress.

The method of applying the cotton is very simple. Raw cotton which is well carded must be selected, in order that it may be free from all foreign substances which it sometimes contains. A layer sufficiently thick to protect the diseased part from the light and air must then be applied, taking care always that the cotton extends some inches beyond the limits of the inflammation. A compress and a few turns of a bandage will keep the cotton applied. A linen mask is well fitted for the face. The cotton should be removed every twenty-four hours to judge of its effects, or, if there is no contra-indication, it may be allowed to remain during the whole course of the treatment. If the cotton should adhere too strongly to the skin, in a case where there is slight exudation, it may be removed by applying over it an emollient poultice. The author has added to the previous remarks, several cases of various forms of erysipelas treated with cotton; to which it is unnecessary to call the attention, otherwise than to state that seven are cases of simple erysipelas of the face and extremities; that one is of traumatic erysipelas; the ninth of erysipelas complicated with a miliary eruption; four of phlegmonous and one of gangrenous erysipelas. If other surgeons meet with the same success which appears to have attended M. Reynaud in the use of his remedy, they will have great reason to thank him for its recommendation.

*Journal des Connaissances Medico-Chirurgicales. Fevrier*, 1837.

---

*Rhinoplastic Operation.* By J. MASON WARREN, M.D., Boston.

[THE following operation is extremely creditable to the judgment and skill of Dr. Warren; and although it cannot now be considered as novel, its perusal cannot fail to be both interesting and instructive to the young surgeon. It is never useless to exhibit the triumphs of surgery to its votaries.]

J. T., twenty-eight years of age. Three years ago last spring, he received a violent blow on the nose, which dislocated the cartilage, driving it at the same time over to the left side. Some inflammation came on in the nose at the time of the accident, which very shortly subsided; and as he was out of town, and at a distance from medical advice, nothing was done to replace the cartilage, which remained in the situation into which it had been driven by the blow. In the following spring, a small red spot appeared on the right cheek just below the eye; this very soon increased in size, the inflammation gradually spread, first attacking the lip, and from thence extending to the nose, which became red, swollen, and finally ulcerated; and in the course of eighteen months the whole nose, cartilages, septum, bones, &c., were successively attacked, and finally completely destroyed. The ulceration had also extended to the cheek of the opposite side. Subsequently to this, cicatrization gradually took place, leaving the patient in the state in which I saw him, six months after his recovery from the disease.

At this period, the nose had entirely disappeared, leaving in the place it originally occupied an opening about an inch in diameter, bordered by a firm cicatrix; the septum of the nostrils was destroyed, and the two nasal cavities thus thrown into one; externally a small cicatrix descended from the lower and left edge of this opening to the angle of the mouth. In the course of the disease the four front teeth had been lost, and this, together with the absorption of the alveolar processes, had caused a sinking of the upper lip, which had fallen an inch below the level of the lower one. An opening also existed between the lip and upper jaw, through which a probe might be passed from the mouth into the nasal cavity. The sense of smel was quite lost, and he was subject to an occasional running of the tears over the face arising from the too sudden contact of the air with the lachrymal ducts.

His case was certainly a hard one. A young man, in the prime of life, in other respects of a good face and appearance, was, by this frightful calamity, not only entirely cut off from society, but prevented from gaining the means of subsistence. Having determined to submit himself to an operation, it was thought expedient to delay it a few weeks, in order to watch the case a little, and prepare him for it by a course of diet and regimen. At the end of six weeks his health had materially improved, and the operation was performed on the 7th of September. A piece of pasteboard, cut in the shape of the letter V, with a projection from its base, corresponding to the columna of the nose, was placed upon the forehead, and a trace made around it with the nitrate of silver. A trace was also made around the opening of the nasal fossa, at the points where it would be necessary to remove the integuments for planting the new skin taken from the forehead. This was done the night previous, in order to prevent any undue delay on the day of the operation. The head being firmly supported by two assistants, the incision was commenced between the eyebrows, and the flap of skin dissected up so as entirely to isolate it from the skin of the forehead, except where, for the purpose of nutrition, it was left adherent at the root of the nose. The incision on the left side between the eyebrows was extended a little farther down than on the right, the better to facilitate the twisting of the flap. This incision included the skin, subcutaneous cellular tissue, and a portion of the occipito-frontalis muscle, care being taken not to raise the periosteum, from fear of necrosis. The flap thus dissected and twisted round to the left side, was carefully wrapped in a compress of linen cloth, and, before the operation was proceeded farther in, attention was given to diminishing the large wound made in the scalp. Little hæmorrhage had taken place, and the temporal arteries, which had been cut, very soon retracted and ceased bleeding. The angles of the wound were first brought together by the twisted suture, two pins being employed on either side. Its edges between the eyebrows were also approximated in a similar manner; by this means the wound in the forehead was diminished at once to less than half its original size; it was still farther reduced by the use of a few strips of adhesive plaster, and a little scraped lint filled up the remainder of the wound. Some lint spread with cerate was spread over the whole surface, a pledget, and the whole secured by a bandage round the head.

The next object was to fix the borrowed skin in its place. For this purpose a short narrow knife, somewhat similar to a cataract knife, was used, and a strip of integument, a third of an inch in breadth, removed, including all that portion which had been at all indurated during the cicatrization of the ulcerations. The knife was also passed between the lip and upper jaw, in which existed, as before stated, an opening large enough to pass a probe, and the adhesions between the two, for the space of an inch, entirely cut away. This was done for the double purpose of giving the columna of the nose a more deep and firm adhesion, and, in the inflammation which would subsequently ensue, to close up the unnatural communication between the mouth and nasal cavity. The flap was now brought down into its place, its angles a little rounded with the scissors, the better to simulate the alæ of the nose, and the whole secured in its place by pins and points of the interrupted suture. From that portion of the skin which was to form the columna of the nose, the epidermic side was pared a little, so that it might form an adhesion not only underneath to the jaw, but on its sides to the quadrangular wound made for it in the upper lip. A little scraped lint was now placed under the ends of the pins, and a strip of oiled lint introduced into each nostril to prevent adhesion; another strip was placed upon the nose to preserve its temperature. The dressings were secured by a band of adhesive plaster fixed to the forehead above, and partially divided in the middle, so that it might descend on each side of the nose to the lip.

During the whole of this long and painful operation the patient kept up his courage, and not a cry was uttered, nor the least struggle made that could at all impede the motions of the operator. Not much blood was lost, and his strength was so little exhausted that he was able to run up stairs to his chamber. He was ordered to go to bed immediately, to keep perfectly quiet, and a watcher left with him, who had directions, in case of his falling asleep, to prevent him from either rolling over on his side, or raising his hand to the nose so as to derange the dressings; also to wake him immediately should he breathe through the nose. To have arrow-root or gruel and lemonade, for nourishment.

September 10th. Passed a good night, slept well. A piece of cork was confined between the teeth, so as to keep the mouth open, it being hoped that this might prevent him from closing his lips during sleep and breathing through the nose.

11th. Quite as well. The introduction of the cork into the mouth had entirely effected its object, by preventing the passage of air through the nose.

12th. The first dressing took place. The dressings on the forehead, after being well soaked, were first removed. The angles of the wound were found to have united throughout, so that two of the pins were at once dispensed with. Union had also taken place in its lower part, just above and between the eyebrows; the remainder of the wound, that is, its central part, in which union by the first intention could not take place, was suppurating well, and filled with healthy granulations. The nose was next attended to. Upon the lint being removed, which had become very much hardened by the coagulated blood, it was found that entire union had taken place on both sides. The alæ of the nose and lower edges could not easily be seen without making use of too much violence in removing the dressings, which at present was not thought necessary. The columna was curved inwards, and the sutures concealed. The nose was of the natural colour and temperature, and the circulation through it seemed uninterrupted. Two strips of lint dipped in oil were laid over the cicatrix on each side of the nose, and no other dressings used. The patient was allowed to sit up a little, and to take any liquid food he might fancy.

One of the pins was removed from the forehead on the 13th, and another, the only remaining one, on the following day. The dossils of lint which had been placed in the nostrils still remained there, firmly caked in by the drying of the pus, blood, &c. These were not removed until the 19th, when their places were supplied by two pieces of hollow sound.

15th. The remaining pins were removed from the side of the nose, and the two sutures which confined the alæ; and on the 17th, ten days after the operation, the two ligatures, which confined the columna in its place, were also removed. At this period, the following was the state of the parts. The wound in the forehead, from the adhesion by the first intention which had taken place, and subsequent contraction had diminished to a third its original size, and the small triangular space which remained, together with that portion of the scalp from which the columna of the nose had been taken, was filled with healthy granulations. From the wound to the root of the nose was a lineal cicatrix two inches in length, and continuous with the cicatrix on the left side. Adhesion of the integuments had taken place on both sides of the nose; at the right alæ, however, the union was not quite so perfect as at the left; that is to say, the whole thickness of the skin did not appear to have united. To assist the union, the skin of the face which lay under it was slightly scarified with the point of a lancet. The columna of the nose was a little curved backward, and its edges had retracted inwards upon themselves. The inside of the nose was suppurating well, and at its upper part adhesion seemed to have taken place between the two bleeding surfaces which had been opposed to each other. The tip of the nose was well defined, and its edges were curved inwards so as well to simulate the natural appearance of the alæ; and just above the alæ, apparently from atmospheric pressure, a depression was taking place, forming their superior boundary. This was assisted by the patient making an occasional pressure with his fingers at these points. He feels well, has a good appetite, and sits up all day. He breathes freely through the tubes placed in the nostrils, which require to be daily removed in order to clear out any obstructions which may collect in them.

At the end of a month the wound in the forehead had contracted to about a quarter of its original size. Adhesion of the nose was perfect at all its points. The openings of the nostrils were regularly rounded, and simulated well the natural appearance. At the end of six weeks he was able to go out and walk about. At the end of two months it was thought time to proceed to the second operation, which was required to remove the twist existing at the root of the nose. The method usually adopted by operators has been to cut the pedicle, after sufficient union of the nose has taken place below to justify the separation of it from its source of nutrition, and to fix it down at the root of the nose, in a transverse incision made for it at that point. To this method there are some serious objections. First, the danger of inflammation in separating the pedicle; second, of sloughing of the organ on the vessels being cut which have hitherto supplied it with blood; and lastly, the very perceptible transverse cicatrix left after the operation. The method resorted to in the present case is liable to none of these objections, except, perhaps, the first one, in which the danger is much diminished. An incision was made, commencing at the internal angle of the eye, and extending to that part of the base of the nose where adhesion had not been able to take place; a corresponding incision was also practised on the pedicle. The skin being well dissected up from its adhesion, a small portion of integument was removed from the upper angle of the wound, where it had become wrinkled from the twist in the pedicle. The edges were brought together by three points of the interrupted suture. The same operation was to be performed at a future day on the other side, where, however, the opening was of about half the size, and not so perceptible. Union took place, throughout, by the first intention. Some trouble was experienced, however, by the formation of a small abscess in the new cicatrix, which suppurated and discharged itself.

Four months after the operation the cicatrization had become complete at all points. The cicatrix in the forehead has become very small, and is gradually assuming the colour of the surrounding integuments. The scalp from which the columna was taken is lost in the hair. The nose is quite firm, of a good form, and the cicatrix on each side hardly perceptible; at the root of the nose on the left side, and at that portion which formed the pedicle, a small fissure still remains, which is for the present concealed by a strip of court plaster. The health of the patient has never been better, his sense of smell is returning, and the tears no longer run over the face, and he, as well as his friends, congratulate themselves both on the moral and physical effects of the operation. He is now able to make his appearance during the daytime, which he has not done before during the last two years, and no person would observe anything remarkable in the nose, without a minute examination, when it would be difficult to explain the remarkable anatomical changes which have taken place.

*Boston Medical and Surgical Journal. March* 8, 1837.

---

### *On the Causes which retard or prevent the Consolidation of Fractures.* By M. Louis Fleury.

[The following observations on the treatment of fracture are the more interesting, from the lively discussions that have recently taken place in this country on the subject. The French author takes no notice of these.]

M. F. remarks that, in order to ensure a perfect consolidation of a fracture, two indications must be fulfilled: 1st. Placing the broken extremities in contact. 2d. Keeping them in this position. The former is done with facility, but the latter, notwithstanding the means resorted to, presents some difficulties. In the present day, most surgeons think that complete immobility of a fractured limb, joined to strong pressure on the soft parts, are the best means of maintaining the fragments in position, and of obtaining a quick and regular consolidation. Unfortunately, these means are frequently unsuccessful; and, notwithstanding the care used in their application, the callus is frequently thrown out in an irregular manner, or perhaps never formed at all. What, then, are the causes which prevent or retard the consolidation of a fracture? Authors have enumerated a great number, but seem to have overlooked a very important one. Scrofulous and venereal affections,—old age,—rents in the periosteum,—formations of pus,—cold applications,—all, undoubtedly, exercise a prejudicial influence. But, by far the most frequent of all the causes, is the apparatus used with the view of favouring the consolidation, which it prevents by the compression it exercises upon the vessels of the limb; whether this compression is inevitable, as in the immoveable apparatus, or produced voluntarily by the surgeon.

If a fractured thigh be placed in a thick layer of soft materials, the effects of the compression cannot be very appreciable; for, in this case, although the capillary circulation and the small arterial branches are more or less restricted, still the large vessels continue free. The same does not occur in the forearm or leg, where compression, ever so slight, interrupts the course of the blood, not only in the superficial vessels, but also in those which supply the fractured bone and periosteum. In order to obtain a rapid and regular consolidation, we must be careful not to apply more splints than are absolutely necessary, and not to bind these too tight by means of bandages. In following an opposite method, we wait sometimes, three, four, or six months for an union which has not yet commenced. It is then that the surgeon, eager at each dressing to reapply the apparatus with more care, that is to say, to augment the number of splints, surround the limb more exactly, &c., finds himself deceived; and the more he renews his efforts, by the same means, the greater is the distance separating him from his object.

The following case, one of four reported, exemplifies the beneficial results of this practice.

CASE. C. D., aged forty-one years, had a fall on the 15th of February, 1836, broke his right leg, and entered the same day the Hospital of St. Louis. The fracture was complete, situated immediately above the internal malleolus, and complicated with a deep excoriation and extensive ecchymosis. The wound was dressed with cerate, the rest of the leg covered with charpie dipped in the white of egg, and the ordinary apparatus for fractures of the leg immediately applied. The member was maintained in absolute repose during six weeks. On the 10th of April, the apparatus was removed, the wound was found cicatrized; the ecchymosis had disappeared, but the consolidation had not commenced. The apparatus was reapplied, and a more generous diet ordered. On the 30th of April, the consolidation was a little more advanced. The splints and anterior cushions were then removed, and the limb sprinkled with spirits of camphor. From this period the callus rapidly solidified; by the end of the month of May, it was very resistant, almost inappreciable to the touch, and the patient quitted the hospital.

*Archives Générales de Médecine. August*, 1837.

---

### THE PROGRESS OF LITHOTRITY.

I. *On the existence of Lithotrity amongst the Arabians during the twelfth and thirteenth Centuries.* By C. F. MARTINS, M.D.

IT appears from several passages in various Arabian writers, that, during the twelfth and thirteenth centuries, they were acquainted to a certain extent with lithotrity. In a work by the celebrated Arabian physician Azzarahvi (vulgarly designated Albucasis, who died at Cordova in 1107,) entitled "*Liber Theorices necnon Practices*," there is the following passage: "Accipiatur instrumentum subtile quod nominant mashafarebilia et suaviter intromittatur in virgam, nunc volve lapidem in media vesicæ, et si fuerit mollis frangitur."

In an Arabian work, entitled "The Flower *of Thoughts* on precious Stones," by Chehab-Eddin-ahmed-Ben-Joussouf-Teifaschy, we have the following observation:—

"One precious advantage of the diamond which Aristotle has spoken of, and which experience confirms, is its employment in calculous affections; when an individual is affected with stone, whether in the bladder or in the urethra, we take a small diamond, and fix it to the extremity of a small stalk of metal, either of copper or silver, and introduce it into the organ containing the stone, which can be pounded by frequent rubbing."

Ahmed-Ben-Abikheled, a physician known by the name of Ebn-al-Harrar, relates, in a work which he has written on calculous disorders, that he employed these means upon a domestic, who suffered from an urinary calculus of a very large size. "This man," says he, "would not submit to the operation of lithotomy; I therefore employed the other means indicated. I pounded the stone by rubbing and reduced it to a size sufficiently small to pass out with the urine."

*Revue Médicale. July*, 1837.

### II. *Improved Lithotritic Instrument.* By M. Civiale.

The instrument which M. Civiale submitted to the Academy of Sciences differs from those which he had hitherto made use of, in the formation of the curved portion. Some difficulty was always experienced in seizing and fixing small stones and fragments of stones, and the operation often caused much fatigue to the patient. M. Civiale has obviated these inconveniences by increasing the surface of the curved part, and diminishing the quantity of material of which it was formed. In the old instrument, the size of the female branch was three lines, and that of the male two lines. In the new instrument the female branch is five lines and a half and the male four, so that the surface by which the calculus is seized is nearly doubled. The instrument is not too much weakened by loss of material, the urethra suffers no injury from increase of size, the bladder is less apt to be pinched, and the stone is less liable to escape. These advantages have been ascertained by experiment, so that we may safely regard the new instrument of M. Civiale as an improvement in the art of lithotrity.

*La Presse Médicale.* Tome i. 1837, p. 479.

### III. *Lithotrity in a Child, aged fourteen Months.* By M. Segalas.

This child was aged fourteen months, and during seven months had manifested symptoms of stone; among others, frequent desire to micturate, violent pain, and particularly whilst making water, almost constant diarrhœa, and prolapsus of the rectum every time that the urine was evacuated. After having detected a calculus, M. Segalas introduced the "brise-pierre," without any other preparation than having dilated the urethra by a wax bougie, allowed to remain for a few minutes in the urethra. A calculus of a diameter of ten lines was seized, and broken by pressure and percussion combined. The calculus, which was composed of oxalate of lime, was entirely destroyed during four sittings. Neither of these was followed by any accident, and during their intervals, the child continued to go to school, to run and play as usual. He subsequently became extremely healthy.

*Bull. de l'Acad. Roy. de Med. July,* 1837.

---

### *On the Separation of the Epiphyses of Bones.* By M. Roux de Brignolle.

The possibility of the separation of the epiphyses of bones after the first few years of life, has been doubted by many eminent surgeons. M. Roux relates a number of cases which prove that these accidents may occur as late as the age of eighteen, and that the largest as well as the smallest bones are liable to them. He relates an instance of separation of the condyles of the femur in a boy of eleven years; of the inferior extremity of the radius, the styloid process of the ulnar, the head of the first phalanx of the thumb, and of the fore and middle fingers of the left hand, in a boy of eighteen years of age; of the humerus in a boy of twelve years; of the lower extremity of both radii in a boy of seventeen; of the head of the humerus in a boy of fifteen; and of the lower extremity of the radius in a man of twenty-two years of age. When the separated portions of bone are not replaced, inflammation, suppuration, intense pain, ardent fever, extensive and deep-seated abscesses, and sometimes even gangrene occur, especially when the larger bones are injured. When the displacement takes place in articulations of moderate size, the separation of the portions of bone is often inconsiderable; but union in that position causes deformity, and impedes and weakens the movements of the joint. In the smallest articulations, the displacement is never great, but, if the accident is neglected, motion is almost impossible, and the deformity very great. The union of the separated portions of bone is very rapid, whether they are restored to their proper place or not.

M. Roux gives the following directions for the treatment of these cases. The separated portions of bone are to be replaced as soon as possible, and retained in their situation by the same means which are resorted to in case of fracture; the treatment, however, does not last so long in separation of the epiphyses. If displacement of the fragments produces pressure on the surrounding parts, and threatens tetanus, paralysis, or gangrene, and if the reduction cannot be effected in the usual way, deep incisions must be made, and the separated portions of bone be either replaced or removed. If the shaft of the bone project through the skin, the opening must be enlarged by incision, or the extremity of the bone removed. M. Roux admits internal as well as external causes of displacement, and terminates his letter by vindicating his claim to originality against M. Guéritin.

*La Presse Médicale.* No. 55. *July* 12, 1837.

---

### *On the Effects of Pus in Contact with Osseous Tissue.*
### By M. MASLIEURAT LAGÉMARD.

THE case here reported was treated in the hospital of the Faculty at Paris, by M. Jules Cloquet. A woman, aged forty-two, was the subject of an abscess in the right axilla; the consequence of which was the formation of fistulous passages, extending upwards towards the clavicle. Whilst under treatment for this affection, she was suddenly attacked with acute inflammation of the left lung, which terminated fatally on the fourth day.

On inspection, a considerable part of the left lung was found infiltrated with pus, whilst the right lung was perfectly healthy and crepitant throughout. Beneath the anterior portion of the great pectoral muscle was discovered a large cavity, extending from the clavicle to the fifth rib, *not* communicating with the fistulous openings, and filled with well-formed pus. The part of the clavicle which was exposed to the action of the pus was denuded of periosteum, and presented a true ulceration with sharp edges, as if a portion of the bone had been removed with a chisel. The first, second, and fourth ribs presented a similar lesion; and in the third rib there was a complete solution of continuity to the extent of seven lines. The corresponding portions of the intercostal muscles were completely destroyed, and the pleura laid bare. This membrane was opaque and excessively thickened, and adhered closely to the opposite portion of the pleura pulmonalis. The adjacent pulmonary tissue was quite healthy.

The reporter supposes that this purulent deposit may have communicated in the first instance with the fistulous openings, and that, this communication being accidentally stopped, the absorption of the different tissues was the result of the consequent retention of the pus. It is worthy of remark, that there were strong adhesions between the pleura pulmonalis and the denuded portion of the pleura costalis, which was not the case elsewhere; so that, had perforation taken place, the pus would have escaped into the bronchi, and a cure might thus have been effected.

We are not acquainted with the conditions which influence the action of pus in the tissues with which it is in contact; nor can we explain why, in this case, the presence of the same fluid should have caused absorption of bone, and condensation of a serous membrane and of cellular tissue. The pus appeared to be well formed, and communication with the external air was prevented. We must therefore look for the specific cause in the peculiar constitution of the individual, and our ignorance on this point should induce us to attend most carefully to the practice, now so generally inculcated, of giving issue to purulent matter as soon as its existence is well ascertained. *Archives génerales de Médecine. Mars,* 1837.

---

### *On the external Use of Calomel in Inflammation of the Eyes.*
### By Dr. FRICKE, of Hamburg.

THE author calls attention to a remedy, now fallen much into disuse, in cases of ophthalmia. He has employed it extensively, and the success with which its application in his practice has been attended makes him anxious to render it better known generally. The first cases which he selected for its use were of a chronic character, and such as had been subjected, without advantage, to other treatment; e. g. rheumatic, catarrhal, and scrofulous inflammations, attended with the various changes which frequently accompany them. Subsequently he employed the remedy in other and milder forms of inflammation; the result of his observations being, that calomel, employed in this manner, is of the greatest utility. In cases of rheumatic inflammation of the eyes, in which, notwithstanding the most rational treatment, local and general means, derivants, narcotics, antirheumatics, &c., the redness, pain, and intolerance of light have continued, the calomel, in the hands of the author, has been attended, in a couple of days, or even after a single application, with an amelioration, or even a disappearance, of all these symptoms. Instead of the great intolerance of light, the eye becomes capable of bearing its stimulus; the burning hot tears become milder and less abundant; the sensation of pain in the eye gradually diminishes, and the patient is soon cured.

The same pleasing results follow the use of calomel in strumous ophthalmia. In the management of that most distressing symptom of this disease, intolerance of light, the author is not aware of one case in which the calomel was applied to the eye, where its application was not followed by success.

In other inflammations of the eyes, in which a general plan of treatment had failed, and in which the calomel was employed experimentally, to observe its action, two only are known to Dr. Fricke in which any injurious effects were produced.

The mode of employing the calomel is as follows:—Dip a miniature pencil into calomel washed in alcohol, and apply it immediately to the eyeball. This application should be repeated once or oftener in the twenty-four hours. The sensations produced in the eye are very different: in a healthy eye, in general, scarcely any sensation is produced, or at most a transient and slight warmth; in inflamed eyes, pain is produced, but it is generally inconsiderable, and only complained of in certain cases. The pain disappears wholly in from half an hour to two hours. One patient felt no pain, but said that the calomel produced a feeling of coldness. Others, to whom Dr. Fricke has communicated the results of his own experience with the use of calomel, have found them in their own practice confirmed.

*Zeitschrift für die gesammte Medicin.* No. 7. *July,* 1837.

---

### *Account of a new Cupping-glass.* By M. Lafargue.

The cupping-glass of M. Lafargue he recommends as obviating the objections which arise from the use of flame, and the expense which attends those to which an exhausting pump is attached: it consists of a glass funnel, and it is exhausted by suction. A small leathern valve at the extremity of the funnel prevents the return of air after it has been withdrawn by the mouth; and so great is the force which may be employed by this simple apparatus, that, "in having recourse to it," says the author, "I produce constantly ecchymosis, and even an exudation of blood on that part of the skin to which I apply it."

When M. Lafargue desires to obtain an energetic revulsion, he takes a disk of cotton or of old linen, holds it before the fire, and, when it has become very hot, he applies it to that part of the skin on which he wishes to operate, places his cupping-glass over, and exhausts it. At the end of two minutes he recommences the operation, which he repeats four or five times successively. It is after the application of cupping instruments that M. Lafargue thinks his instrument is of special advantage. He has no doubt that, by the use of his apparatus, as much blood may be obtained as by the use of leeches.

[This is the aboriginal instrument of the cow's horn improved by modern science. We doubt not but it is very efficacious.]

*Gazette Médicale.* No. 25. *Juin,* 1836.

---

### *Curious Case of a Silver Teaspoon swallowed during a Paroxysm of Madness.* By Dr. Otto, of Copenhagen.

The subject of this case endeavoured in various ways to put an end to his life. After attempting it by fourteen days' starvation, and finding that this did not answer his purpose, he swallowed a silver spoon, thinking that this would ensure his destruction. This was on the 21st July, 1835. He was subject to such treatment as would, as much as possible, prevent the occurrence of inflammation. A constant pain was complained of in the scrobiculus cordis, which pain had commenced after swallowing the spoon, and compelled the individual to lean forwards constantly. The pain continued without relief; palliatives only being given from the conviction that its cause was the presence of the spoon. All his insanity had ceased. In April, 1836, he remarked a small swelling in the præcordia, which broke, and discharged a clear fluid. July 23rd, he observed something dark coloured in this swelling, which was the handle of the spoon. The spoon was withdrawn; the pain ceased, and the ulcer from which the spoon escaped soon cicatrized. *Zeitschrift für die gesammte Medicin.* No. 7. *July*, 1837.

---

*Ossification of the Vitreous Humour.* By Professor ANT. GRILLO.

THIS case formed the subject of a paper read to the Acad. Med. Chir. of Naples, in March, 1837.

A young sailor, whose father was gouty, began to suffer from gout at the age of twenty-seven, attended with disease of the urinary organs, and the passage of much phosphate of lime in his water. His health was improved by proper diet and the use of diuretics; but, about two years afterwards, his sight grew weak, and, with slight ophthalmia, he suffered severe pain in the eyeballs and forehead; his urine being clear, and he being free from gout. An attempt was made to restore the gout to its former seat, but in vain, and he died of apoplexy.

The gouty parts were found enlarged, and loaded with phosphate of lime; as were also the kidneys, which were hypertrophied. The membranes of the brain were inflamed, and the eyeballs were smaller than usual, hard and swelled on each side, with a total loss of transparency. The sclerotic was hard, but could be separated from the parts beneath; but the iris had contracted adhesions to the cornea and to the lens, which was diminished in volume and opaque. The black matter of the choroid, which was confounded with the internal surface of the sclerotic, was alone distinguishable; all the internal part of the eye being occupied by a hard osseous body, in which were confused together the vitreous humour, the hyaloid, the retina, and the membrana Jacobiana.

This case, rare as it is, is not quite unparalleled; for, in August last, Dr. Casilli met with a similar morbid appearance in the eyes of a phthisical subject, and the specimen was laid before the Academy by Dr. Grillo, in confirmation of his own case. *Filiatre Sebazio.* *April*, 1837

---

*Extraction of a foreign Body from the Female Bladder, by artificial Dilatation of the Urethra.* By M. THOMAS.

A LADY, aged thirty-four years, was seized, without any apparent cause, with retention of urine. Her husband, thinking to relieve her, introduced an ivory ear-picker into the urethra, with immediate relief; but, the quantity of urine appearing exceedingly small, he reintroduced the same instrument more deeply. It, however, slipped from his fingers, and fell into the bladder. From that moment symptoms peculiar to stone in the bladder manifested themselves. M. T. was called in six hours after. With a sound he felt the foreign body, and thought to extract it by a small pair of polypus forceps; but failed. The patient suffered much, and passed some blood. An operation was proposed, and objected to. A sponge tent was then introduced into the urethra, and confined by ligatures. In the course of two hours it was swollen considerably by the absorption of the urine; this was then removed, and replaced by a larger. Two hours after, the index finger was passed into the bladder, and the foreign body was felt, placed transversely to its neck: the position being rectified, it was immediately expelled. The urine flowed involuntarily for six hours, but from that time it was under the influence of the will, and the patient recovered.

*La Lancette Française.* *March*, 1837.

